# Dual-fluorescent reporter for live-cell imaging of the ER during DENV infection

**DOI:** 10.3389/fcimb.2022.1042735

**Published:** 2022-10-25

**Authors:** Lochlain Corliss, Madeline Holliday, Nicholas J. Lennemann

**Affiliations:** Department of Microbiology, University of Alabama at Birmingham, Birmingham, AL, United States

**Keywords:** flavivirus, dengue virus, Zika virus, positive strand RNA virus, reporter, live-cell, yellow fever virus (YFV)

## Abstract

Infection by flaviviruses leads to dramatic remodeling of the endoplasmic reticulum (ER). Viral replication occurs within virus-induced vesicular invaginations in the ER membrane. A hallmark of flavivirus infection is expansion of the ER membrane which can be observed at specific time points post infection. However, this process has not been effectively visualized in living cells throughout the course of infection at the single cell resolution. In this study, we developed a plasmid-based reporter system to monitor flavivirus infection and simultaneous virus-induced manipulation of single cells throughout the course of infection in real-time. This system requires viral protease cleavage to release an ER-anchored fluorescent protein infection reporter that is fused to a nuclear localization signal (NLS). This proteolytic cleavage allows for the translocation of the infection reporter signal to the nucleus while an ER-specific fluorescent marker remains localized in the lumen. Thus, the construct allows for the visualization of virus-dependent changes to the ER throughout the course of infection. In this study, we show that our reporter was efficiently cleaved upon the expression of multiple flavivirus proteases, including dengue virus (DENV), Zika virus (ZIKV), and yellow fever virus (YFV). We also found that the DENV protease-dependent cleavage of our ER-anchored reporter exhibited more stringent cleavage sequence specificity than what has previously been shown with biochemical assays. Using this system for long term time-lapse imaging of living cells infected with DENV, we observed nuclear translocation of the reporter signal beginning approximately 8 hours post-infection, which continued to increase throughout the time course. Interestingly, we found that increased reporter signal translocation correlated with increased ER signal intensity, suggesting a positive association between DENV infection and ER expansion in a time-dependent manner. Overall, this report demonstrates that the FlavER platform provides a useful tool for monitoring flavivirus infection and simultaneously observing virus-dependent changes to the host cell ER, allowing for study of the temporal nature of virus-host interactions.

## Introduction

Flaviviruses belong to a large family of enveloped viruses with positive-sense single-stranded RNA genomes. The most prominent human viruses in this family are arthropod-borne and transmitted mainly through the bite of an infected mosquito (Valderrama et al., 2017; Weaver et al., 2018; Souza-Neto et al., 2019; Spadar et al., 2021). The prevalence of the arthropod vector of flaviviruses is the main contributor to the sporadic epidemic outbreaks in geographic locations with high mosquito populations. Members of this viral family cause a significant global health concern, with yellow fever virus (YFV) responsible for ~ 200,000 cases per year and dengue virus (DENV) causing an overwhelming ~ 390 million annual cases (Simo et al., 2019; Pierson and Diamond, 2020; Gaythorpe et al., 2021). Infection with flaviviruses can lead to disease resulting in severe clinical manifestations in multiple organ systems of the human body. Disease symptoms can include hemorrhagic fever or shock, caused by DENV and YFV, encephalitis, caused by West Nile virus (WNV), and birth defects such as microcephaly as a result of Zika virus (ZIKV) infection in pregnant women (Rossi et al., 2010; Duarte et al., 2017; Uno and Ross, 2018). Flavivirus epidemics have devastating effects on human health as well as significant social and economic burdens to the affected regions (Daep et al., 2014; Guo et al., 2017; Hameed et al., 2021). There are few safe and effective vaccines and no approved antiviral treatments for diseases caused by flaviviruses (Heinz and Stiasny, 2012; Ishikawa et al., 2014; Thomas and Yoon, 2019). Given the world-wide prevalence of flavivirus infections, it is crucial to continue the development of tools that can aid in obtaining a more in-depth understanding of the molecular processes of these pathogens that lead to manipulation of the host cell to advance the development of effective preventions and treatments.

The endoplasmic reticulum (ER) is the largest organelle of the cell and serves an array of important cellular functions, including production, trafficking, and degradation of proteins, metabolism of carbohydrates, and synthesis of lipids, making it an optimal site for flavivirus replication (Fagone and Jackowski, 2009; Braakman and Hebert, 2013; Ruggiano et al., 2014; Schwarz and Blower, 2016; Rajah et al., 2020). After viral entry of the host cell through endocytosis, the viral genome is directly translated as a single polyprotein that is embedded into the host cell ER through multiple transmembrane domains (Mazeaud et al., 2018; Ngo et al., 2019). The polyprotein is then co- and post-translationally processed by viral and host proteases into ten individual subunits, composed of three structural proteins and seven nonstructural (NS) proteins (Amberg and Rice, 1999). The viral protease is made up of a complex between NS2B and NS3 (NS2B3). NS3 contains a serine protease domain that requires interaction with the ER-anchored NS2B protein *via* a cytoplasmic cofactor domain in order to be catalytically active (Falgout et al., 1991; Yusof et al., 2000; Xing et al., 2020). Thus, the active viral protease complex resides at the ER membrane in infected cells and is responsible for all cytoplasmic polyprotein cleavage events at a conserved motif, dibasic residues followed by Gly/Ser/Ala/Thr (Chambers et al., 1990; Chambers et al., 1991; Shiryaev et al., 2007). Transmission electron microscopy has revealed remarkable manipulation of the ER during flavivirus replication, including the formation of replication vesicles that concentrate viral and host factors required for replication and packaging (Welsch et al., 2009; Gillespie et al., 2010; Paul, 2013; Junjhon et al., 2014; Nagy et al., 2016; Lennemann and Coyne, 2017; Arakawa and Morita, 2019; Evans et al., 2020). Several nonstructural proteins, such as the heavily membrane-associated NS4A and NS4B, have been shown to lead to drastic rearrangements of the ER membrane; however, expression of these proteins alone is not sufficient for the formation of replication vesicles (Kaufusi et al., 2014; Liang et al., 2016). Thus, viral manipulation of the ER is an important aspect during infection; however, the mechanisms and timing of this critical event are not well understood.

While the process of ER membrane remodeling has been studied utilizing the traditional method of imaging fixed cells, visualization of the events of virus-induced host cell manipulation throughout the course of infection has not been well-characterized. Imaging of fixed samples only allows for still snapshots of different cells at specific time points. However, the process of host cell manipulation during infection is very dynamic; therefore, still images cannot provide the full picture of the events that take place throughout the course of infection. To overcome these limitations, long-term time-lapse imaging of living cells infected with recombinant viruses expressing reporter fluorescent proteins has been used in previous reports (Schoggins et al., 2012; Shang et al., 2013; van der Schaar et al., 2016). However, genetic manipulation of the flavivirus genome to insert a reporter gene was shown to cause substantial attenuation of viral progeny. To bypass this obstacle, other studies have constructed plasmid-based reporter systems that rely on viral protease-dependent cleavage to release the fluorescent reporter signal to translocate to the nucleus and indicate infection (Jones et al., 2010; Medin et al., 2015; McFadden et al., 2018; Pahmeier et al., 2021). The limitation of these constructs is that they are able to detect only the presence of infection and cannot allow for the visualization of virus-induced changes to the host cellular structures. We previously developed a plasmid-based reporter to use in concert with live-cell imaging to detect infection by enteroviruses and track virus-induced manipulation of the host cell simultaneously (Lennemann et al., 2020). Therefore, we sought to expand on this construct by tailoring it to detect flavivirus infection and subsequent ER manipulation. In this study, we validate the efficacy of this reporter construct, termed FlavER, and uncover the temporal correlation between DENV infection and ER expansion.

## Materials and methods

### Cell culture and viruses

U2OS osteosarcoma cells (ATCC, HTB-96) and HEK-293T cells (ATCC, CRL-11268) were cultured in Dulbecco’s modified Eagle’s medium supplemented with 10% FBS and 100 IU penicillin/100 µg/mL streptomycin. Vero E6 cells (a gift from Dr. Kevin Harrod, University of Alabama at Birmingham) were cultured in modified Eagle’s medium supplemented with 10% fetal bovine serum and 100 IU penicillin/100 µg/mL streptomycin. C6/36 cells were cultured in Dulbecco’s modified Eagle’s medium supplemented with 10% FBS and 100 IU penicillin/100 µg/mL streptomycin. Human hepatoma cells (Huh7) were maintained in DMEM supplemented with 10% FBS, 9 g/L glucose, and 100U/mL P/S. All mammalian cells were maintained at 37°C in a humidified environment with 5% CO_2_. C6/36 cells were maintained in a humidified environment at 28°C.

Dengue virus serotype 2 strain 16681 (a gift from Carolyne Coyne, Duke University) was propagated in C6/36 cells (ATCC, CRL-1660) incubated at 33°C for 6 days. Supernatants were combined with FBS to a final concentration of 20%, and cell debris was removed through centrifugation at 2300 xg for 15 minutes at 4°C. Clarified supernatants were aliquoted and stored at -80°C for future use. Yellow fever virus strain 17D was obtained from BEI Resources (NR-116) and propagated in VeroE6 cells for 4 days. Supernatants were clarified and aliquoted prior to titration *via* focus forming assay. Human astrovirus-1 strain Oxford (BEI Resources) was propagated and titrated by focus forming assay as previously described (Marvin et al., 2014).

### Focus forming assay

Flavivirus stocks were titered by focus forming assay as previously described with minor modifications (Payne et al., 2006). Briefly, Vero cells were incubated with serial dilutions of virus for 48 hours. Cells were fixed in 4% paraformaldehyde (PFA; Electron Microscopy Sciences) diluted in phosphate buffered saline (PBS; Corning, #21-040-CM) for 10 minutes at room temperature (RT), permeabilized in ice-cold methanol for 5 minutes at RT, and washed in PBS. Monolayers were incubated with α-flavivirus E-protein monoclonal antibody (clone 4G2, a gift from Margaret Kielian, Albert Einstein College of Medicine) for 1 hour at RT, washed with PBS, and incubated with secondary α-mouse antibody conjugated to Alexa Fluor 488 (Invitrogen, A11029) for 30 minutes at RT followed by a 5-minute incubation with 300 nM 4′,6-diamidino-2-phenylindole (DAPI; Invitrogen) diluted in PBS. Foci were counted using an Olympus IX83 inverted fluorescent microscope.

### Plasmid construction

FlavER was cloned through the assembly of three DNA fragments into the NheI and XbaI restriction sites of pcDNA3.1(+) (Invitrogen). The first fragment was generated by amplifying the GFP from pcDNA3.1-GFP (a gift from Carolyn Coyne, Duke University) using Q5 high-fidelity DNA polymerase (New England Biolabs) with NheI-GFP_F (5’-GAAGCTAGCCACCATGGTGAGCAAGGGCGAGGAG-3’) and Xhol-GFP_R primers (5’-GAGACTCGAGCTTGTACAGCTCGTCCATGC-3’), followed by digestion with NheI-HF and XhoI (New England Biolabs). The second fragment was synthesized as a gBlock (Integrative DNA Technologies) consisting of 5’-XhoI – simian virus 40 nuclear localization sequence (-TCATCCGATGACGAGGCCACAGCTGATTCCCAGCACTCAACTCCGCCTAAAAAAAAAAGAAAAGTT) – KpnI – a flavivirus protease consensus cleavage sequence (GCAGAGGCTGCAAAAAGGAGGAGTGCTGGACTGAACGAGATG) – BamHI – transferrin receptor transmembrane domain (TATGGGACTATTGCTGTGATCGTCTTTTTCTTGATTGGATTTATGATTGGCTACTTGGGCTAT) – flexible linker (GGTGGATCTGGCGGAGGTTCCGGC) – EcoRI-3’, which was digested with XhoI and EcoRI-HF (New England Biolabs). The third fragment was generated by amplifying the mCherry-KDEL from pAc-mCherry-KDEL (a gift from Carolyn Coyne, Duke University) using Q5 high-fidelity DNA polymerase with EcoRI-mCh_F (5’-GTAGGGAATTCATGGTGAGCAAGGGCGAGGAGGATAAC-3’) and XbaI-mCh-KDEL_R (5’-GTAACGTTAGGGGGGGGGGATCTAGATCATAGCTCGTCTTTCTTGTACAGC-3’) to generate an EcoRI and XbaI flanked mCherry-KDEL. The three fragments were digested with the respective terminal restriction sites and ligated using T4 DNA ligase (New England Biolabs) for 3 hours at RT. The ligation was transformed into DH5α *E. coli* (Zymo Research).

pLJM1entr was generated to facilitate cloning of FlavER into pLJM1, a lentivirus transfer plasmid. The EGFP transgene in pLJM1-EGFP (a gift from was a gift from David Sabatini (Addgene plasmid # 19319, (Sancak et al., 2008) was replaced at the AgeI and EcoRI restriction sites with the following annealed oligos: Entr_F (5’- CCGGTTAATACGACTCACTATAGGGGATATCCCTCGACTGTGCCTTCTAG-3’) and Entr_R (5’- AATTCTAGAAGGCACAGTCGAGGGATATCCCCTATAGTGAGTCGTATTAA-3’) containing an EcoRV restriction sequence flanked by sequences homologous to the 5’ and 3’ untranslated regions of pcDNA3.1(+). pLJM1-FlavER was generated through the amplification of FlavER using Q5 high-fidelity DNA polymerase and pLJM1entry_F (5’-GAACCGTCAGATCCGCTAGCTAATACGACTCAC TATAGGG-3’) and pLJM1entry_R (5’-CATTTGTCTCGAGGTCGAGAATTCCACAGT CGAGGCTGATCAGC-3’) primers. This PCR product was assembled with EcoRV linearized pLJM1entr using HiFi DNA Assembly (New England Biolabs) according to the manufacturer’s instructions. This reaction was transformed into NEB Stable *E. coli*.

Expression plasmids for DENV NS2B-3-V5, DENV NS2B-3-S135A-V5, ZIKV NS2B-3-V5, and WNV NS2B-3-V5 have been previously described (Lennemann and Coyne, 2017). To construct YFV NS2B-3-V5, a plasmid expressing the yellow fever protease sequence was ordered from Twist Bioscience. This plasmid was then digested with NotI and KpnI restriction enzymes and ligated into the pcDNA3.1(+) vector expressing a V5 epitope tag.

### Immunofluorescence microscopy

U2OS cells (40,000 cells/well) were reverse transfected with FlavER plasmid using Fugene HD (Promega) in an 8-well chamber slide (Celltreat). Twenty-four hours post transfection, cells were fixed in 4% PFA in PBS, permeabilized with 0.1% Triton X-100 (Fisher) diluted in PBS, washed with PBS, and incubated with mouse α -V5 epitope tag monoclonal antibody (Invitrogen, 46-0705) or anti-dsRNA monoclonal antibody (Kerafast) for 1 hour at RT. The monolayers were washed 3X with PBS for 5 minutes each and probed with goat α -mouse conjugated to Alexa Fluor-647 (Invitrogen, A21236) for 30 minutes at RT. The cells were washed 3X with PBS for 5 minutes each and incubated in PBS containing 300 nM DAPI for 5 minutes at RT. The slide was mounted using Vectashield Antifade Mounting Media (Vector Laboratories) and a 24x50mm Premium Superslip (FisherScientific). Three fields of view per well were captured using an Olympus IX83 inverted fluorescent microscope.

### Immunoblots

U2OS cells were transfected with the indicated plasmids using XtremeGene360 (Roche). When specified, cells were infected with DENV for the indicated timepoints. Cells were lysed in 1x RIPA + protease inhibitor + 0.1% SDS cocktail (Sigma). Lysates were sonicated prior to being separated by SDS PAGE using a 4-20% Tris-glycine polyacrylamide pre-cast gel (BioRad) and transferred to nitrocellulose membranes. Following 30 min of blocking in PBS + 10% non-fat milk, membranes were probed with the indicated primary antibodies: mouse anti-V5 (Invitrogen), rabbit anti-GFP (Proteintech), mouse anti-GFP (Proteintech), mouse anti-actin (Proteintech, rabbit anti-actin (Proteintech), and rabbit anti-DENV NS3 (GeneTex) followed by near-infrared dye-conjugated secondary antibodies (LiCor) diluted in PBST + 5% non-fat milk and imaged on an Odyssey CLx imaging system (LiCor).

### Lentivirus production and transductions

To produce lentiviral vectors expressing the FlavER transgene, HEK-293T cells (2 x 10^6^ cells/well) were reverse transfected with 1 µg pLJM1-FlavER, 0.75 µg psPAX2 (a gift from Didier Trono (Addgene plasmid #12260), and 0.25 µg pCAGGS-G-Kan (a gift from Todd Green, University of Alabama at Birmingham) using Lipofectamine 3000 Transfection Reagent (Invitrogen) according to the manufacturer’s instructions. Lentivirus was harvested by passing the media through 0.2-micron filters (Thermo Scientific, 725-2520) 48 hours post transfection. U2OS cells (5 x 10^4^ cells) were reverse transduced with 50 µL FlavER lentiviral vector stock in growth media containing 10 µg/mL polybrene (Sigma Aldrich). Transduced cells were passaged for 48 hours and selected with 5 µg/mL puromycin for 5 days.

### Long-term time-lapse fluorescent live-cell imaging

3 x 10^4^ U2OS cells stably expressing FlavER were transferred to an 8-well live-imaging slide (Ibidi). Cells were either left uninfected (mock) or infected with DENV at an MOI of 10. Infections were synchronized at 4°C for 1h, followed by shifting cells to 37°C for 3h prior to onset of imaging. One image per well was taken every 20 minutes over a 20-hour time period. Image series were cropped using ImageJ software (NIH, Bethesda, MA, USA), and multi-panel images were constructed using Photoshop CC 2021 (Adobe, San Jose, CA, USA).

### Image and data analysis

Models were made using Adobe Illustrator/Photoshop and Biorender. Percent nuclear translocation of the reporter signal in cells transfected with FlavER and a V5-tagged flavivirus protease was quantified using the counter plugin in ImageJ (NIH). The ImageJ counter plugin was also used to identify the number of the protease expressing cells with the reporter signal in the nucleus. Data was plotted using Prism 9 software (GraphPad, San Diego, CA, USA).

ImageJ was used to quantify relative infection signal from the live-cell time-lapse images. To account for non-specific fluorescence signal in the nucleus from the widefield microscope, the fluorescence signal intensity of the infection reporter was determined relative to the ER signal intensity within the same circular region of interest within the nucleus of individual cells from every image series. Data was plotted using Prism 9 software (GraphPad).

### Statistics

One-way ANOVA and nonlinear regression curve analyses were performed using Prism Software 9 software (GraphPad).

## Results

### Flavivirus reporter plasmid construction

**Figure 1:**
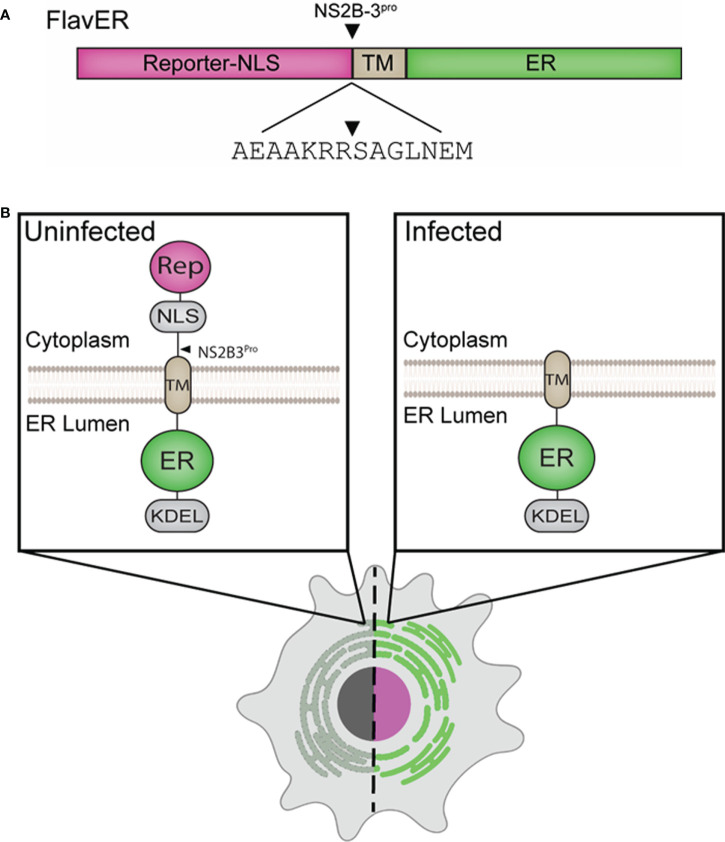
Design of an ER and flavivirus infection reporter. **(A)** Linear model of FlavER. The FlavER reporter consists of a fluorescent protein infection reporter fused to a nuclear localization sequence (NLS) followed by a consensus viral protease (NS2B3) cleavage sequence fused to a transmembrane domain (TM) followed by a fluorescent protein ER marker. Black triangle represents the site cleaved by the viral protease. **(B)** Proposed model of FlavER function. In uninfected cells (left), the reporter signal and ER marker colocalize at the ER. During flavivirus infection (right), viral NS2B3 cleavage releases the reporter signal, which then translocates to the nucleus.

To characterize flavivirus infection-dependent manipulation of host cell organelles in real-time, we modified our previously reported bipartite enterovirus reporter to include a flavivirus protease cleavage motif termed FlavER (**Figure 1**) (Lennemann et al., 2020). It was hypothesized that this plasmid-based reporter system would allow for simultaneous monitoring of flavivirus infection and virus-induced changes to the ER. FlavER was constructed to express a cytoplasmic fluorescent protein fused to a simian virus 40 nuclear localization signal and the flavivirus NS2B3 protease cleavage sequence obtained through multiple sequence alignment of all cytoplasmic cleavage sequences processed by the viral protease within the DENV polyprotein (**Figure 1A**). This fusion protein is anchored to the ER membrane *via* the transmembrane domain of the transferrin receptor (type II transmembrane domain) fused to an ER lumen localized fluorescent protein, which is fused to an ER retention signal (KDEL). Thus, we anticipated that the expression of the NS2B3 protease upon flavivirus infection results in cleavage of the consensus cleavage site, allowing for nuclear translocation of the reporter protein from the ER. Post cleavage, the fluorescent ER protein marker will remain in the ER lumen, allowing for visualization of changes to this organelle throughout the course of infection (**Figure 1B**).

### Validation of FlavER

**Figure 2:**
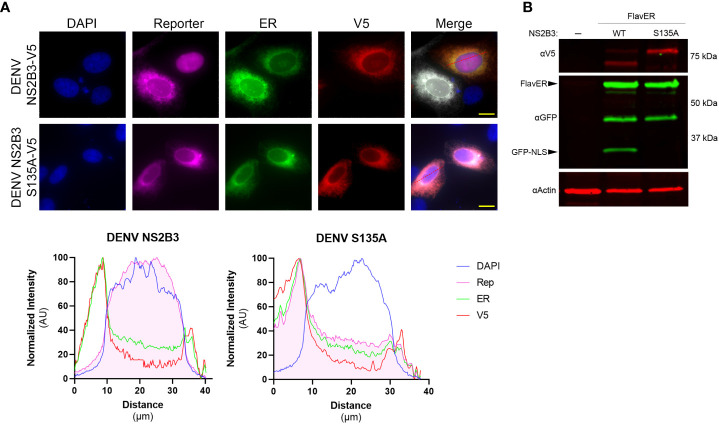
FlavER validation with DENV NS2B3. (**A**, top panel) Immunofluorescence (IF) microscopy of U2OS cells transfected with FlavER and an empty vector or V5-tagged DENV NS2B3. Reporter is shown in magenta, ER marker is shown in green, V5 staining is shown in red, and nuclei are stained with DAPI (blue). Scale bars represent 20 μm. (A, bottom panel) Line plot analyses of normalized signal intensities of DAPI (blue), reporter (magenta), ER (green), and V5 staining (red) for the IF images in the top panel. **(B)** Immunoblot for V5, GFP, and actin of U2OS cells stably expressing FlavER transfected with an empty vector, active DENV protease, or inactive DENV protease (S135A). Labeled arrows indicate the full-length FlavER construct and the cleaved construct (GFP-NLS). *, denotes an intermediate product due to the non-specific proteolysis of mCherry, which has been previously reported (Huang et al., 2014).

To determine if FlavER could be cleaved by the dengue virus protease, U2OS cells were co-transfected with FlavER and V5 epitope-tagged active DENV NS2B3 (WT) or catalytically inactive DENV NS2B3 (S135A) and subjected to immunofluorescence (IF) staining or immunoblotting. IF staining of cells expressing FlavER and the active DENV NS2B3 showed localization of the protease-dependent reporter signal in the nucleus, while cells transfected with the catalytically inactive DENV protease (S135A) did not demonstrate this translocation (**Figure 2A**, top). Intensity profile analysis of IF images showed that cells transfected with the active DENV protease had much higher reporter signal intensity in the nuclear region as compared to the inactive protease control (**Figure 2A**, bottom). Consistently, immunoblot analysis of cell lysates revealed that expression of the DENV NS2B3 protease led to the production of a cleavage fragment representing the separation of GFP-NLS from the full-length FlavER construct. This cleavage fragment was absent in cells expressing the mutant DENV protease, which is unable to self-cleave at the NS2B/NS3 junction, resulting in slower migration of this protease in the gel (**Figure 2B**).

**Figure 3:**
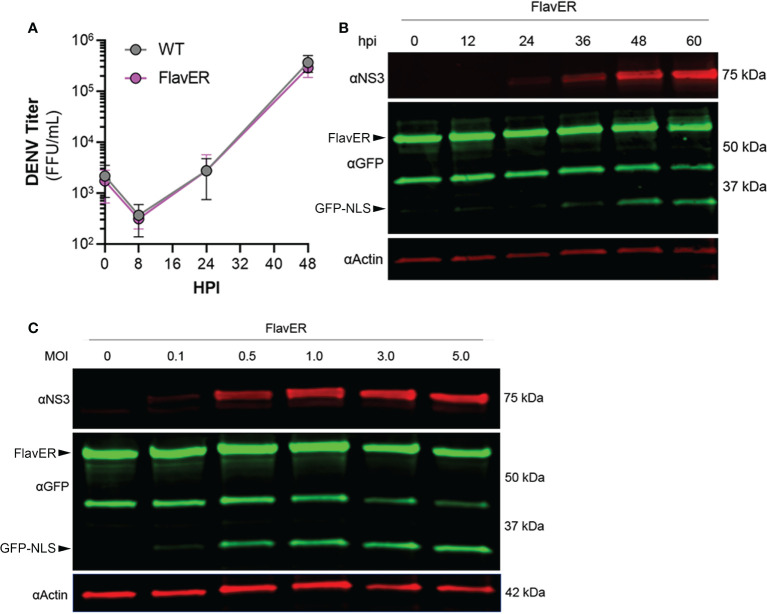
FlavER validation with DENV infection. **(A)** Growth kinetics of DENV in wildtype and FlavER-expressing Huh7 cells. Huh7 cells were infected with DENV at an MOI of 0.1, and FFU assays were performed on supernatants collected at the indicated time points, n=3. Data points represent the average titer (FFU/mL) for three independent experiments. **(B)** Immunoblot for NS3, GFP, and actin of U2OS cells stably expressing FlavER, infected with DENV at an MOI of 0.1, and lysed at the indicated hour post infection. **(C)** Immunoblot for NS3, GFP, and actin of U2OS cells stably expressing FlavER and infected with the indicated MOI of DENV. Cell lysates were harvested 24 hours post infection.

We next wanted to test the efficacy of FlavER with DENV infection, so we first determined if FlavER expression resulted in viral attenuation. Titration of supernatants collected from WT and stable FlavER-expressing cells infected with DENV (0.1 FFU/cell) indicated no difference in viral production over 48 hours of infection (**Figure 3A**). To assess the utility of FlavER during infection, we performed immunoblot analysis on lysates from DENV-infected cells expressing FlavER that were lysed at increasing hours post infection (**Figure 3B**). We also analyzed lysates from FlavER-expressing cells that were infected with an increasing multiplicity of infection (MOI) of DENV (**Figure 3C**). Both experiments revealed an increase of the cleavage fragment that was proportionate to the expression of DENV NS3 during the course of infection. Together, these results demonstrate the efficacy of FlavER in detecting protease activity upon expression of DENV NS2B-3 *via* transfection or infection.

**Figure 4:**
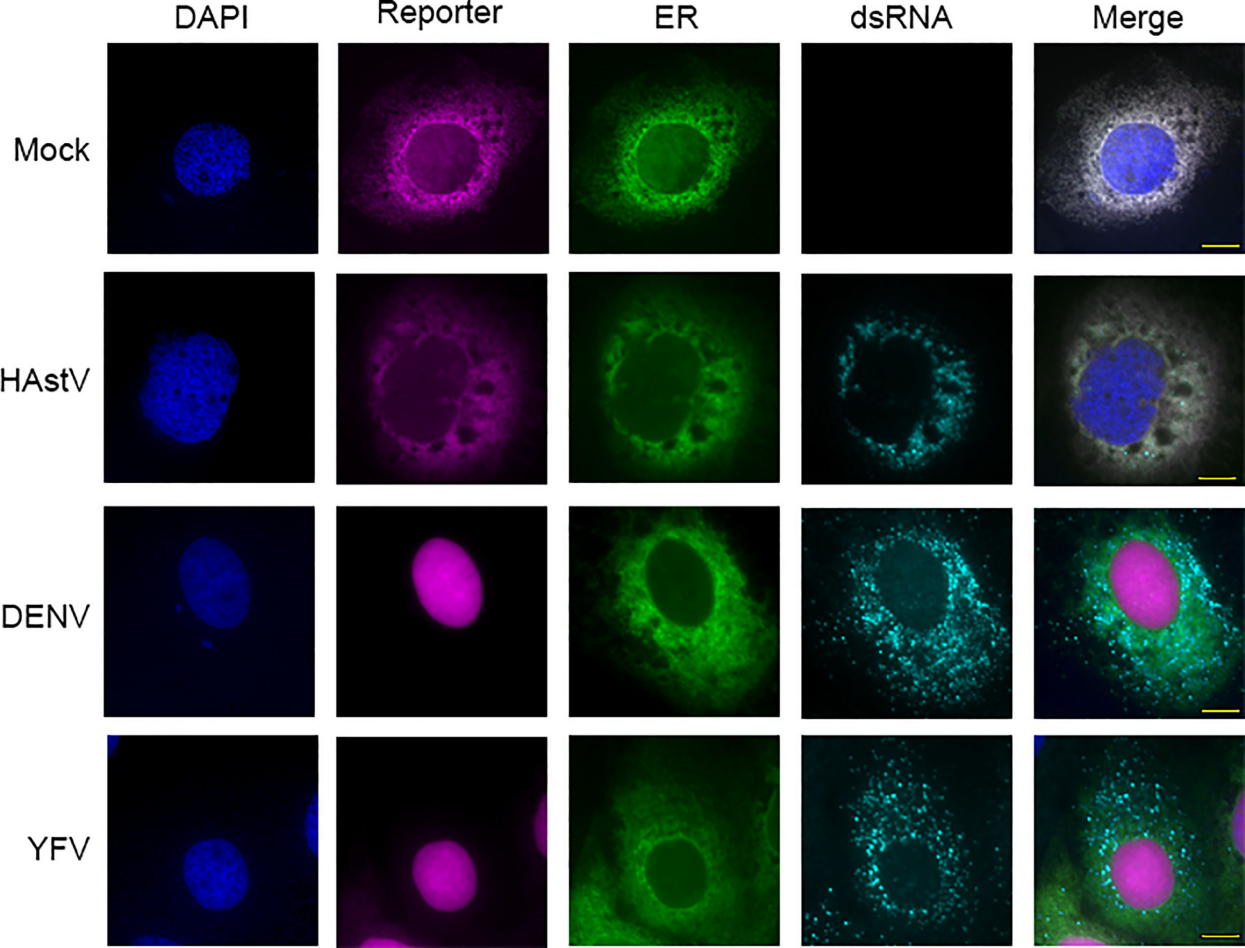
FlavER cleavage is specific to flavivirus infection. Immunofluorescence microscopy of Huh7 cells expressing FlavER and infected with the indicated virus (MOI=3). Reporter is shown in magenta, ER marker is shown in green, dsRNA staining is shown in red, and nuclei are stained with DAPI (blue). Cells were fixed and stained 24 hours post infection.

To determine if FlavER reporter activity was specific to flavivirus infection, Huh7 cells expressing FlavER were infected with DENV, YFV, or human astrovirus (HAstV), an unrelated positive sense ssRNA virus that also replicates at the ER (**Figure 4**) (Guix et al., 2004; Fuentes et al., 2011). After 24 hours, cells were fixed and stained for double-stranded RNA (dsRNA), an RNA intermediate that occurs during active replication of RNA viruses. We found that while both flavivirus infections resulted in efficient nuclear translocation of the reporter signal, infection with HAstV did not, suggesting that the consensus sequence within FlavER is specific for flavivirus proteases (**Figure 4**). Furthermore, FlavER is applicable for multiple flavivirus infections.

### Multiple flavivirus proteases cleave FlavER

**Figure 5:**
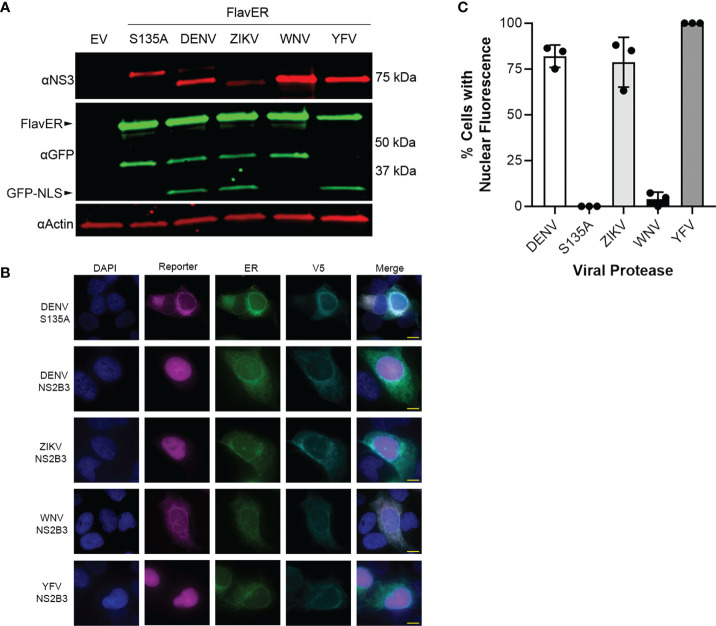
FlavER is cleaved by multiple flavivirus proteases. **(A)** Immunoblot for NS3, GFP, and actin for U2OS cells co-transfected with FlavER and the indicated flavivirus protease. **(B)** Immunofluorescence microscopy images of U2OS cells co-transfected with FlavER and the indicated V5-tagged protease. Reporter is shown in magenta, ER marker is shown in green, V5 staining is shown in cyan, and nuclei are stained with DAPI (blue). Scale bars represent 20 μm. **(C)** Quantification of IF images of cells expressing FlavER and the indicated V5-tagged viral protease. Data is presented as efficiency of cleavage determined by the percentage of co-expressing cells with nuclear-translocated fluorescent infection reporter, n=3. Data points represent results from three different fields of view, with the averages shown as bar graphs.

To determine if the consensus sequence in FlavER could be cleaved by multiple flavivirus proteases, we analyzed the lysates of FlavER-expressing cells transfected with the ZIKV, WNV, YFV, and mutant and active DENV proteases (**Figure 5A**). Immunoblots showed FlavER cleavage by the DENV, ZIKV, and YFV proteases, but not by the WNV or mutant DENV proteases. These findings were corroborated by IF staining of cells under the same conditions (**Figure 5B**). We then quantified immunofluorescence images for the presence of the infection reporter in the nucleus of cells co-transfected with FlavER and DENV, DENV (S135A), ZIKV, WNV, or YFV NS2B3 (**Figure 5C**). DENV, ZIKV, and YFV NS2B3 expression led to the efficient cleavage and release of the reporter signal with ~82%, ~79%, and 100% nuclear translocation, respectively. WNV NS2B3 was the least efficient in cleaving the reporter at ~4% nuclear translocation, whereas no nuclear translocation of the reporter was observed with the catalytically inactive DENV NS2B3. Together, these data show the efficient cleavage of FlavER by DENV, ZIKV, and YFV proteases and the lack of cleavage performed by the WNV protease.

### DENV protease has more strict specificity for intracellular substrates

**Figure 6:**
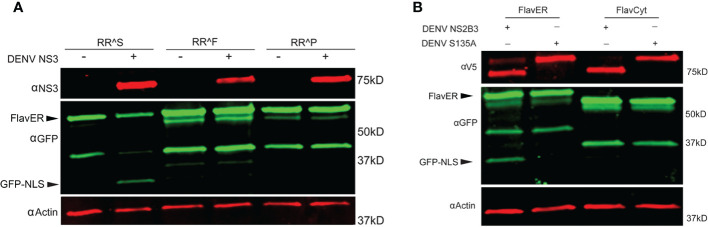
FlavER shows intracellular substrate specificity for the DENV protease. **(A)** Immunoblot for NS3, GFP, and actin for U2OS cells expressing the indicated FlavER construct and either left uninfected (mock) or infected with DENV at an MOI of 3. RRS, RRF, and RRP correspond to the residues in the P2/P1/P1’ position of the FlavER consensus sequence. **(B)** Immunoblot of V5, GFP, and actin for U2OS cells expressing FlavER or FlavCyt and transfected with the indicated DENV protease.

To better understand the applications of FlavER, we sought to determine how an intracellular reporter system differs from biochemical assays in terms of substrate specificity for the DENV protease. A previous study using biochemical assays with a soluble DENV protease and short peptide substrates showed that their protease was 90-100% effective at cleaving substrates with a phenylalanine and a proline as the residue positioned immediately after the dibasic residues in the cleavage motif (P1’), which are not naturally observed in the flavivirus polyprotein sequence (Shiryaev et al., 2007). We used site-directed mutagenesis to introduce single amino acid mutations to the P1’ site of our original FlavER construct, encoding a serine, to express a phenylalanine and a proline. U2OS cells expressing our WT and site-directed mutant reporters were infected with DENV at an MOI of 3 and lysed after 24 hours. Immunoblot analysis revealed that only the WT reporter with a serine in the P1’ position was capable of being cleaved by the DENV protease (**Figure 6A**). Because all but one protease cleavages in the polyprotein occur proximal to the ER membrane, and the active protease localizes to the ER membrane, we sought to determine whether ER membrane-association of the substrate was a molecular determinant for cleavage. We constructed another mutant reporter from our original FlavER template where we removed the TM domain and the NLS of FlavER to make a soluble, protease-dependent cleavage reporter, termed FlavCyt due to its localization in the cytoplasm. U2OS cells were co-transfected with the active or mutant DENV protease and FlavER or FlavCyt. Immunoblot analysis of lysates showed that only the WT FlavER was able to be cleaved while FlavCyt was not, suggesting that reporter localization is a determinant for the ER membrane-associated DENV NS2B3 protease. (**Figure 6B**). Together, these data highlight several determinants for studying the intracellular activity of the flavivirus protease.

### DENV infection leads to reporter translocation to the nucleus

**Figure 7:**
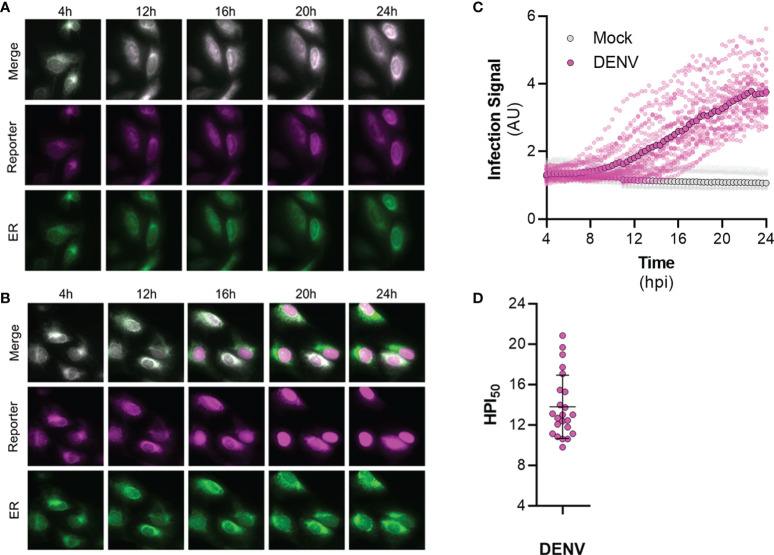
Time-lapse imaging of DENV infected cells expressing FlavER. **(A, B)** Representative time-points of an image series from live-cell imaging of the reporter signal (magenta), the ER marker (green), and merged panels from mock **(A)** or DENV infected **(B)** U2OS cells expressing FlavER. Scale bars represent 20 μm. **(C)** Quantification of infection intensity, defined as the ratio of reporter to ER fluorescence intensity signals within the nucleus from individual mock (gray, n=12) or DENV infected (magenta, n=23) U2OS cells expressing FlavER. Images were taken every 20 minutes, and infection intensity was calculated for every frame throughout a 20-hour time-lapse imaging experiment. Individual data points represent the average reporter intensity at the indicated timepoint. **(D)** Determination of the timepoint at which the 50% maximum reporter signal was observed in the nucleus (HPI50) as determined by nonlinear regression analysis for individual DENV-infected cells (n=23).

To validate the efficacy of FlavER during infection, long-term time-lapse imaging was used to determine the efficiency of virus-induced nuclear translocation of the reporter signal. U2OS cells stably expressing FlavER were infected with DENV four hours prior to being placed in a humidified chamber set to 37°C for a 20-hour timespan with images taken in 20-minute intervals. DENV infection of cells expressing the reporter resulted in evident translocation of the fluorescent protein-NLS reporter to the nucleus, which was not observed in mock cells, while the ER-KDEL signal was shown to remain localized in the lumen throughout the course of a 24-hour infection (**Supplemental Movies 1**, **2**; **Figures 7A, B**). Individual cells were quantified to determine the intensity of the reporter signal relative to the ER signal in the nucleus over time. Analysis of the image series showed that infected cells displayed a linear increase in nuclear-localized reporter signal throughout the course of infection (**Figure 7C**). Uninfected (mock) cells exhibited no increase in nuclear reporter signal. Further analysis revealed that reporter translocation occurred in DENV-infected cells starting at ~8 hours post infection, and 50% of the maximum signal was reached at ~14 hours post infection on average (**Figure 7D**). Taken together, these results suggest that the FlavER reporter can be used to detect infection *via* protease activity throughout the course of infection.

### DENV infection correlates with ER expansion in a time-dependent manner

**Figure 8:**
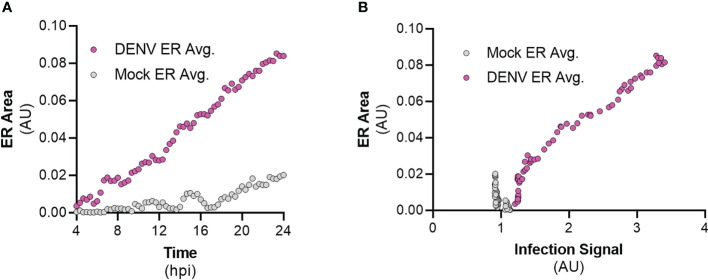
DENV infection correlates with ER expansion in a time-dependent manner. **(A)** Quantification of ER intensity for mock (gray, n=12) and DENV-infected cells (magenta, n=14) over the course of a 24-hour infection. **(B)** Correlation analysis between ER intensity and infection for mock (gray, n=12) and DENV-infected cells (magenta, n=14).

To understand the kinetics of ER expansion as a result of infection by flaviviruses, we performed live-cell imaging of FlavER-expressing cells infected with DENV and quantified the resulting image series for changes in ER signal intensity over time. Cells infected with DENV showed a clear increase in the perinuclear ER signal intensity over the 20-hour imaging period, while uninfected cells showed no such increase (**Figure 8A**). We also wanted to observe the relationship between infection and ER expansion, so we performed a correlation analysis which revealed that cells infected with DENV show a strong positive correlation between the infection reporter signal in the nucleus and perinuclear ER signal intensities (**Figure 8B**). Together, these data show that as infection with DENV progresses, there is a quantifiable increase in perinuclear ER signal intensity.

## Discussion

Long-term time-lapse imaging of living cells is a valuable method for studying virus-host interactions during the course of infection. In this study, this technique was used to monitor virus-induced changes to the ER of the host cell using a bipartite fluorescent reporter that requires flavivirus protease cleavage to release the NLS-tagged fluorescent protein. Release of this protein allows for its translocation to the nucleus of the host cell, while the ER-localized fluorescent marker is retained in the lumen. Additionally, we have shown that this system is a valuable tool for studying the determinants of flavivirus protease activity at the ER membrane, which is where it localizes during infection. Overall, this study shows that the design of the FlavER reporter provides a platform for understanding the temporal nature of virus-host interactions and protease activity during infection in real-time.

Previous groups have designed reporter viruses with a fluorescent tag inserted into the genomes of flaviviruses (Zou et al., 2011; Schoggins et al., 2012; Shang et al., 2013; Gadea et al., 2016; Suphatrakul et al., 2018; Tamura et al., 2018; Torres et al., 2022). These reporter viruses are useful in identifying infected cells; however, they do not accurately recapitulate the viral replication kinetics due to the attenuation that occurs when introducing a reporter gene into the genome. We have shown that our plasmid-based reporter system bypasses the issue of viral attenuation in cells expressing FlavER (**Figure 3A**). Other studies have also employed plasmid-based reporter systems for live-cell imaging to monitor flavivirus infection in host cells. These groups designed their flavivirus infection reporters with the fluorescent reporter signal fused to viral non-structural protein 4B (NS4B) (Jones et al., 2010; Medin et al., 2015; McFadden et al., 2018). However, flavivirus NS4B is a multi-pass ER membrane-anchored protein that has been previously shown to induce autophagy, cause remodeling of the ER, and down-regulation of interferon-stimulating genes in cells expressing this protein alone (Munoz-Jordan et al., 2003; Kaufusi et al., 2014; Liang et al., 2016). These effects can make the results of experiments using these constructs difficult to interpret, which can be avoided using the FlavER system that has no impact on viral infection.

More recently, other groups have constructed plasmid-based reporters also dependent on viral protease activity; however, these reporters are limited to detecting infection and do not allow for the visualization of host structures unless additional markers are co-expressed in cells (Arias-Arias et al., 2020; Pahmeier et al., 2021). Previously, we designed a dual-fluorescent enterovirus reporter construct that allows for detection of infection and simultaneous visualization of host cell organelles (Evans et al., 2020). We then adapted this system to a single plasmid that can monitor flavivirus infection while observing the host cell ER in parallel. Therefore, FlavER improves upon previous constructs in that the design eliminates the overexpression of viral proteins known to manipulate the host cell and the need for transfection of multiple plasmids to simultaneously monitor infection and host cell manipulation. With the advantage of the ER-anchored fluorescent protein fused to the infection reporter, we were able to observe the changes that occurred in the ER throughout the course of DENV infection. Using live-cell imaging, we observed a quantifiable increase in ER signal intensity that positively correlated with the increase in infection (**Figures 8A, B**). This report uncovered the temporal nature of the relationship between DENV infection and virus-induced expansion of the ER. This correlation was unable to be shown previously with traditional methods of fixed cell microscopy, further demonstrating the usefulness of live-cell imaging to observe virus-induced manipulation of organelles at the single cell resolution.

Our reporter system not only allows us to monitor infection and ER manipulation in living cells but also provides a platform to investigate flavivirus protease specificity for intracellular substrates. First, we found that while the DENV, ZIKV, and YFV proteases are capable of cleaving the same consensus cleavage sequence, the WNV protease had very poor cleavage efficiency, which is consistent with a previous report (Shiryaev et al., 2007) (**Figures 5B, C**). These results suggest that different flavivirus proteases have distinct cleavage preferences for certain residues in intracellular substrates. Thus, future studies using FlavER can elucidate the differences for substrate preference and cleavage efficiency for multiple flavivirus proteases. Further, we determined that the DENV protease was unable to cleave versions of FlavER encoding Phe or Pro residues in the P1’ position (**Figure 6A**). These results contradict what has been shown in previous biochemical studies, suggesting there are differences in protease activity in intracellular systems (Shiryaev et al., 2007). Additionally, our reporter system revealed that substrate localization to the ER is another molecular determinant for flavivirus protease cleavage (**Figure 6B**), which is supported by the ER membrane-proximal cleavage sites present in the viral polyprotein.

Overall, this study demonstrates the efficacy of our bipartite plasmid-based reporter to monitor viral infection and simultaneously observe virus-induced manipulation of the host cell in real-time, while also showing the importance of studying viral protease specificity in the context of the cell. Because this reporter construct allows for the indication of viral infection by distinct nuclear translocation of the infection signal to the nucleus, this system can be applied to high-throughput screens to identify antiviral therapeutics. By simply modifying the viral cleavage recognition sites, our reporter can be adapted for other positive-strand RNA viruses, including alphaviruses and coronaviruses. With the FlavER design, it is also possible to engineer the construct in a way that builds a multipartite reporter that allows for the visualization of virus-induced changes to multiple host cell structures. The reporter can be expanded for the observation of infection-induced changes to multiple host cell organelles simultaneously, such as the Golgi complex, mitochondria, lipid droplets, or the cytoskeleton by modifying the construct to contain three or even four fluorescent organelle marker proteins.

## Data availability statement

The original contributions presented in the study are included in the article/**Supplementary Material**. Further inquiries can be directed to the corresponding author.

## Author contributions

Conceptualization: LC, MH, and NL. Methodology: LC, MH, and NL. Validation: LC, MH, NL. Formal analysis: LC, and NL. Investigation: LC, and NL. Resources: NL. Data curation: LC, and NL. Writing—original draft preparation: LC. Writing—review and editing: LC, and NL. Visualization: LC, and NL. Supervision: NL. Project administration: NL. Funding acquisition: NL. All authors have read and agreed to the published version of the manuscript. All authors contributed to the article and approved the submitted version.

## Funding

This project was supported by developmental funds from University of Alabama at Birmingham Department of Microbiology (NL) and NIH grant K22AI143963-01 (NL).

## Acknowledgments

We thank Carolyne Coyne (Duke University), Kevin Harrod (University of Alabama at Birmingham), and Todd Green (University of Alabama at Birmingham) for reagents.

## Conflict of interest

The authors declare that the research was conducted in the absence of any commercial or financial relationships that could be construed as a potential conflict of interest.

## Publisher’s note

All claims expressed in this article are solely those of the authors and do not necessarily represent those of their affiliated organizations, or those of the publisher, the editors and the reviewers. Any product that may be evaluated in this article, or claim that may be made by its manufacturer, is not guaranteed or endorsed by the publisher.

## Supplementary material

Supplemental Movie 1 Live-cell imaging of individual DENV infected U2OS cells expressing FlavER. Movies were taken over a 20-hour timespan with images captured every 20 minutes.

Supplemental Movie 2 Live-cell imaging of individual uninfected cells expressing FlavER. Movies were taken over a 20-hour timespan with images captured every 20 minutes.

## References

[B1] Amberg S. M. Rice C. M. (1999). Mutagenesis of the NS2B-NS3-Mediated cleavage site in the flavivirus capsid protein demonstrates a requirement for coordinated processing. J. Virol. 73, 8083–8094. doi: 10.1128/JVI.73.10.8083-8094.1999 10482557 PMC112824

[B2] Arakawa M. Morita E. (2019). Flavivirus replication organelle biogenesis in the endoplasmic reticulum: Comparison with other single-stranded positive-sense RNA viruses. Int. J. Mol. Sci. 20 (9), 2336. doi: 10.3390/ijms20092336 PMC6539296 31083507

[B3] Arias-Arias J. L. MacPherson D. J. Hill M. E. Hardy J. A. Mora-Rodríguez R. (2020). A fluorescence-activatable reporter of flavivirus NS2B–NS3 protease activity enables live imaging of infection in single cells and viral plaques. J. Biol. Chem. 295, 2212–2226. doi: 10.1074/jbc.RA119.011319 31919100 PMC7039541

[B4] Braakman I. Hebert D. N. (2013). Protein folding in the endoplasmic reticulum. Cold Spring Harb. Perspect. Biol. 5, a013201–a013201. doi: 10.1101/cshperspect.a013201 23637286 PMC3632058

[B5] Chambers T. J. Grakoui A. Rice C. M. (1991). Processing of the yellow fever virus nonstructural polyprotein: a catalytically active NS3 proteinase domain and NS2B are required for cleavages at dibasic sites. J. Virol. 65, 6042–6050. doi: 10.1128/jvi.65.11.6042-6050.1991 1833562 PMC250270

[B6] Chambers T. J. Weir R. C. Grakoui A. McCourt D. W. Bazan J. F. Fletterick R. J.. (1990). Evidence that the n-terminal domain of nonstructural protein NS3 from yellow fever virus is a serine protease responsible for site-specific cleavages in the viral polyprotein. Proc. Natl. Acad. Sci. 87, 8898–8902. doi: 10.1073/pnas.87.22.8898 2147282 PMC55067

[B7] Daep C. A. Muñoz-Jordán J. L. Eugenin E. A. (2014). Flaviviruses, an expanding threat in public health: focus on dengue, West Nile, and Japanese encephalitis virus. J. Neurovirol. 20, 539–560. doi: 10.1007/s13365-014-0285-z 25287260 PMC4331079

[B8] Duarte G. Moron A. Timerman A. Fernandes C. Mariani Neto C. Almeida Filho G.. (2017). Zika virus infection in pregnant women and microcephaly. Rev. Bras. Ginecologia e Obstetrícia / RBGO Gynecol. Obstetrics 39, 235–248. doi: 10.1055/s-0037-1603450 PMC10316959 28575919

[B9] Evans A. S. Lennemann N. J. Coyne C. B. (2020). BPIFB3 regulates endoplasmic reticulum morphology to facilitate flavivirus replication. J. Virol. 94(9), e00029–20. doi: 10.1128/JVI.00029-20 32102874 PMC7163128

[B10] Fagone P. Jackowski S. (2009). Membrane phospholipid synthesis and endoplasmic reticulum function. J. Lipid Res. 50, S311–S316. doi: 10.1194/jlr.R800049-JLR200 18952570 PMC2674712

[B11] Falgout B. Pethel M. Zhang Y. M. Lai C. J. (1991). Both nonstructural proteins NS2B and NS3 are required for the proteolytic processing of dengue virus nonstructural proteins. J. Virol. 65, 2467–2475. doi: 10.1128/jvi.65.5.2467-2475.1991 2016768 PMC240601

[B12] Fuentes C. Guix S. Bosch A. Pintó R. M. (2011). The c-terminal nsP1a protein of human astrovirus is a phosphoprotein that interacts with the viral polymerase. J. Virol. 85, 4470–4479. doi: 10.1128/jvi.01515-10 21325412 PMC3126255

[B13] Gadea G. Bos S. Krejbich-Trotot P. Clain E. Viranaicken W. El-Kalamouni C.. (2016). A robust method for the rapid generation of recombinant zika virus expressing the GFP reporter gene. Virology 497, 157–162. doi: 10.1016/j.virol.2016.07.015 27471954

[B14] Gaythorpe K. A. Hamlet A. Jean K. Garkauskas Ramos D. Cibrelus L. Garske T.. (2021). The global burden of yellow fever. Elife 10, e64670. doi: 10.7554/eLife.64670 33722340 PMC7963473

[B15] Gillespie L. K. Hoenen A. Morgan G. Mackenzie J. M. (2010). The endoplasmic reticulum provides the membrane platform for biogenesis of the flavivirus replication complex. J. Virol. 84, 10438–10447. doi: 10.1128/JVI.00986-10 20686019 PMC2950591

[B16] Guix S. Caballero S. Bosch A. Pintó R. M. (2004). C-terminal nsP1a protein of human astrovirus colocalizes with the endoplasmic reticulum and viral RNA. J. Virol. 78, 13627–13636. doi: 10.1128/jvi.78.24.13627-13636.2004 15564473 PMC533902

[B17] Guo C. Zhou Z. Wen Z. Liu Y. Zeng C. Xiao D.. (2017). Global epidemiology of dengue outbreaks in 1990–2015: A systematic review and meta-analysis. Front. Cell Infect. Microbiol. 7. doi: 10.3389/fcimb.2017.00317 PMC5506197 28748176

[B18] Hameed M. Wahaab A. Shan T. Wang X. Khan S. Di D.. (2021). A metagenomic analysis of mosquito virome collected from different animal farms at yunnan–Myanmar border of China. Front. Microbiol. 11. doi: 10.3389/fmicb.2020.591478 PMC7898981 33628201

[B19] Heinz F. X. Stiasny K. (2012). Flaviviruses and flavivirus vaccines. Vaccine 30, 4301–4306. doi: 10.1016/j.vaccine.2011.09.114 22682286

[B20] Huang L. Pike D. Sleat D. E. Nanda V. Lobel P. (2014). Potential pitfalls and solutions for use of fluorescent fusion proteins to study the lysosome. PLoS One 9, e88893. doi: 10.1371/journal.pone.0088893 24586430 PMC3931630

[B21] Ishikawa T. Yamanaka A. Konishi E. (2014). A review of successful flavivirus vaccines and the problems with those flaviviruses for which vaccines are not yet available. Vaccine 32, 1326–1337. doi: 10.1016/j.vaccine.2014.01.040 24486372

[B22] Jones C. T. Catanese M. T. Law L. M. J. Khetani S. R. Syder A. J. Ploss A.. (2010). Real-time imaging of hepatitis c virus infection using a fluorescent cell-based reporter system. Nat. Biotechnol. 28 (2), 167–71. doi: 10.1038/nbt.1604 20118917 PMC2828266

[B23] Junjhon J. Pennington J. G. Edwards T. J. Perera R. Lanman J. Kuhn R. J. (2014). Ultrastructural characterization and three-dimensional architecture of replication sites in dengue virus-infected mosquito cells. J. Virol. 88, 4687–4697. doi: 10.1128/JVI.00118-14 24522909 PMC3993787

[B24] Kaufusi P. H. Kelley J. F. Yanagihara R. Nerurkar V. R. (2014). Induction of endoplasmic reticulum-derived replication-competent membrane structures by West Nile virus non-structural protein 4B. PLoS One 9 (1), e84040. doi: 10.1371/journal.pone.0084040 24465392 PMC3896337

[B25] Lennemann N. J. Coyne C. B. (2017). Dengue and zika viruses subvert reticulophagy by NS2B3-mediated cleavage of FAM134B. Autophagy 13 (2), 322–332. doi: 10.1080/15548627.2016.1265192 28102736 PMC5324851

[B26] Lennemann N. J. Evans A. S. Coyne C. B. (2020). Imaging-based reporter systems to define CVB-induced membrane remodeling in living cells. Viruses 12 (10), 1074. doi: 10.3390/v12101074 PMC7600424 32992749

[B27] Liang Q. Luo Z. Zeng J. Chen W. Foo S.-S. Lee S.-A.. (2016). Zika virus NS4A and NS4B proteins deregulate akt-mTOR signaling in human fetal neural stem cells to inhibit neurogenesis and induce autophagy. Cell Stem Cell 19 (5), 663–671. doi: 10.1016/j.stem.2016.07.019 27524440 PMC5144538

[B28] Marvin S. Meliopoulos V. Schultz-Cherry S. (2014). Human astrovirus propagation, purification and quantification. Bio Protoc. 4 (6), e1078. doi: 10.21769/bioprotoc.1078

[B29] Mazeaud C. Freppel W. Chatel-Chaix L. (2018). The multiples fates of the flavivirus RNA genome during pathogenesis. Front. Genet. 9. doi: 10.3389/fgene.2018.00595 PMC6288177 30564270

[B30] McFadden M. Mitchell-Dick A. Vazquez C. Roder A. Labagnara K. McMahon J.. (2018). A fluorescent cell-based system for imaging zika virus infection in real-time. Viruses 10 (2), 95. doi: 10.3390/v10020095 PMC5850402 29495257

[B31] Medin C. L. Valois S. Patkar C. G. Rothman A. L. (2015). A plasmid-based reporter system for live cell imaging of dengue virus infected cells. J. Virol. Methods 211, 55–62. doi: 10.1016/j.jviromet.2014.10.010 25445884 PMC4342618

[B32] Munoz-Jordan J. L. Sanchez-Burgos G. G. Laurent-Rolle M. Garcia-Sastre A. (2003). Inhibition of interferon signaling by dengue virus. Proc. Natl. Acad. Sci. 100 (24), 14333–14338. doi: 10.1073/pnas.2335168100 14612562 PMC283592

[B33] Nagy P. D. Strating J. R. P. M. van Kuppeveld F. J. M. (2016). Building viral replication organelles: Close encounters of the membrane types. PloS Pathog. 12 (10), e1005912. doi: 10.1371/journal.ppat.1005912 27788266 PMC5082816

[B34] Ngo A. M. Shurtleff M. J. Popova K. D. Kulsuptrakul J. Weissman J. S. Puschnik A. S. (2019). The ER membrane protein complex is required to ensure correct topology and stable expression of flavivirus polyproteins. Elife 8, e48469. doi: 10.7554/eLife.48469 31516121 PMC6756788

[B35] Pahmeier F. Neufeldt C. J. Cerikan B. Prasad V. Pape C. Laketa V.. (2021). A versatile reporter system to monitor virus-infected cells and its application to dengue virus and SARS-CoV-2. J. Virol. 95 (4), e01715–20. doi: 10.1128/JVI.01715-20 33257477 PMC7851548

[B36] Paul D. Bartenschlager R. (2013). Architecture and biogenesis of plus-strand RNA virus replication factories. World J. Virol. 2 (2), 32–48. doi: 10.5501/wjv.v2.i2.32 24175228 PMC3785047

[B37] Payne A. F. Binduga-Gajewska I. Kauffman E. B. Kramer L. D. (2006). Quantitation of flaviviruses by fluorescent focus assay. J. Virol. Methods 134 (1–2), 183–189. doi: 10.1016/j.jviromet.2006.01.003 16510196

[B38] Pierson T. C. Diamond M. S. (2020). The continued threat of emerging flaviviruses. Nat. Microbiol. 5, 796–812. doi: 10.1038/s41564-020-0714-0 32367055 PMC7696730

[B39] Rajah M. M. Monel B. Schwartz O. (2020). The entanglement between flaviviruses and ER-shaping proteins. PLoS Pathog. 16, e1008389. doi: 10.1371/journal.ppat.1008389 32298390 PMC7161947

[B40] Rossi S. L. Ross T. M. Evans J. D. (2010). West Nile Virus. Clin. Lab. Med. 30, 47–65. doi: 10.1016/j.cll.2009.10.006 20513541 PMC2905782

[B41] Ruggiano A. Foresti O. Carvalho P. (2014). ER-associated degradation: Protein quality control and beyond. J. Cell Biol. 204, 869–879. doi: 10.1083/jcb.201312042 24637321 PMC3998802

[B42] Sancak Y. Peterson T. R. Shaul Y. D. Lindquist R. A. Thoreen C. C. Bar-Peled L.. (2008). The Rag GTPases bind raptor and mediate amino acid signaling to mTORC1. Science 320 (5882), 1496–501. doi: 10.1126/science.1157535 18497260 PMC2475333

[B43] Schoggins J. W. Dorner M. Feulner M. Imanaka N. Murphy M. Y. Ploss A.. (2012). Dengue reporter viruses reveal viral dynamics in interferon receptor-deficient mice and sensitivity to interferon effectors *in vitro* . Proc. Natl. Acad. Sci. 109 (36), 14610–14615. doi: 10.1073/pnas.1212379109 22908290 PMC3437900

[B44] Schwarz D. S. Blower M. D. (2016). The endoplasmic reticulum: structure, function and response to cellular signaling. Cell. Mol. Life Sci. 73 (1), 79–94. doi: 10.1007/s00018-015-2052-6 26433683 PMC4700099

[B45] Shang B. Deng C. Ye H. Xu W. Yuan Z. Shi P.-Y.. (2013). Development and characterization of a stable eGFP enterovirus 71 for antiviral screening. Antiviral Res. 97 (2), 198–205. doi: 10.1016/j.antiviral.2012.12.010 23267829

[B46] Shiryaev S. A. Kozlov I. A. Ratnikov B. I. Smith J. W. Lebl M. Strongin A. Y. (2007). Cleavage preference distinguishes the two-component NS2B–NS3 serine proteinases of dengue and West Nile viruses. Biochem. J. 401 (3), 743–752. doi: 10.1042/BJ20061136 17067286 PMC1770841

[B47] Simo F. B. N. Bigna J. J. Kenmoe S. Ndangang M. S. Temfack E. Moundipa P. F.. (2019). Dengue virus infection in people residing in Africa: a systematic review and meta-analysis of prevalence studies. Sci. Rep. 9, 13626. doi: 10.1038/s41598-019-50135-x 31541167 PMC6754462

[B48] Souza-Neto J. A. Powell J. R. Bonizzoni M. (2019). Aedes aegypti vector competence studies: A review. Infection Genet. Evol. 67, 191–209. doi: 10.1016/j.meegid.2018.11.009 PMC8135908 30465912

[B49] Spadar A. Phelan J. E. Benavente E. D. Campos M. Gomez L. F. Mohareb F.. (2021). Flavivirus integrations in aedes aegypti are limited and highly conserved across samples from different geographic regions unlike integrations in aedes albopictus. Parasit Vectors 14, 332. doi: 10.1186/s13071-021-04828-w 34174947 PMC8235865

[B50] Suphatrakul A. Duangchinda T. Jupatanakul N. Prasittisa K. Onnome S. Pengon J.. (2018). Multi-color fluorescent reporter dengue viruses with improved stability for analysis of a multi-virus infection. PloS One 13, e0194399. doi: 10.1371/journal.pone.0194399 29547653 PMC5856417

[B51] Tamura T. Fukuhara T. Uchida T. Ono C. Mori H. Sato A.. (2018). Characterization of recombinant flaviviridae viruses possessing a small reporter tag. J. Virol. 92 (2), e01582–17. doi: 10.1128/JVI.01582-17 29093094 PMC5752933

[B52] Thomas S. J. Yoon I.-K. (2019). A review of dengvaxia®: development to deployment. Hum. Vacc. Immunother. 15, 2295–2314. doi: 10.1080/21645515.2019.1658503 PMC6816420 31589551

[B53] Torres F. J. Parry R. Hugo L. E. Slonchak A. Newton N. D. Vet L. J.. (2022). Reporter flaviviruses as tools to demonstrate homologous and heterologous superinfection exclusion. Viruses 14, 1501. doi: 10.3390/v14071501 35891480 PMC9317482

[B54] Uno N. Ross T. M. (2018). Dengue virus and the host innate immune response. Emerg. Microbes Infect. 7, 1–11. doi: 10.1038/s41426-018-0168-0 30301880 PMC6177401

[B55] Valderrama A. Díaz Y. López-Vergès S. (2017). Interaction of flavivirus with their mosquito vectors and their impact on the human health in the americas. Biochem. Biophys. Res. Commun. 492, 541–547. doi: 10.1016/j.bbrc.2017.05.050 28499872

[B56] van der Schaar H. M. Melia C. E. van Bruggen J. A. Strating J. R. van Geenen M. E. Koster A. J.. (2016). Illuminating the sites of enterovirus replication in living cells by using a split-GFP-Tagged viral protein. mSphere 1 (4), e00104–16. doi: 10.1128/mSphere.00104-16 PMC4935779 27390781

[B57] Weaver S. C. Charlier C. Vasilakis N. Lecuit M. (2018). Zika, chikungunya, and other emerging vector-borne viral diseases. Annu. Rev. Med. 69, 395–408. doi: 10.1146/annurev-med-050715-105122 28846489 PMC6343128

[B58] Welsch S. Miller S. Romero-Brey I. Merz A. Bleck C. K. E. Walther P.. (2009). Composition and three-dimensional architecture of the dengue virus replication and assembly sites. Cell Host Microbe 5, 365–375. doi: 10.1016/j.chom.2009.03.007 19380115 PMC7103389

[B59] Xing H. Xu S. Jia F. Yang Y. Xu C. Qin C.. (2020). Zika NS2B is a crucial factor recruiting NS3 to the ER and activating its protease activity. Virus Res. 275, 197793. doi: 10.1016/j.virusres.2019.197793 31676367

[B60] Yusof R. Clum S. Wetzel M. Murthy H. M. K. Padmanabhan R. (2000). Purified NS2B/NS3 serine protease of dengue virus type 2 exhibits cofactor NS2B dependence for cleavage of substrates with dibasic amino acids in vitro. J. Biol. Chem. 275, 9963–9969. doi: 10.1074/jbc.275.14.9963 10744671

[B61] Zou G. Xu H. Y. Qing M. Wang Q.-Y. Shi P.-Y. (2011). Development and characterization of a stable luciferase dengue virus for high-throughput screening. Antiviral Res. 91, 11–19. doi: 10.1016/j.antiviral.2011.05.001 21575658

